# Soluble Programmed Death-1 Predicts Clinical Outcome After STEMI

**DOI:** 10.1016/j.jacbts.2024.03.001

**Published:** 2024-05-27

**Authors:** Ahmad Hayek, Camille Brun, Simon Leboube, Florentin Moulin, Nathan Mewton, Gabriel Bidaux, Sylvie Ducreux, Mélanie Paillard, Claire Crola Da Silva, Thomas Bochaton

**Affiliations:** a INSERM, INRA, INSA Lyon, Université Claude Bernard Lyon 1, Groupement Hospitalier Est, Bron, France; b Hôpital Louis Pradel, Hospices Civils de Lyon, Bron, France

In the aftermath of ST-segment elevation myocardial infarction (STEMI), an inflammatory response occurs, and it is now considered an important prognostic element and a predictor of mortality. Programmed death (PD)-1 is an inhibitory receptor of the CD28 family and is expressed on antigen-activated T lymphocytes. PD-1 inhibits T-cell receptor kinase-dependent signals after binding with its ligands.^1^ It is considered as a checkpoint protein, down-regulating effector T cells and maintaining immune tolerance.^2^ We hypothesized that PD-1, through its immune exhaustion function, may have a role at the acute phase of STEMI. Our aim was to determine the plasma level of soluble PD (sPD)-1 after STEMI and its relation with infarct size and clinical events.

We conducted a prospective monocentric study including 286 patients from 2016 to 2020. The study was approved by our ethics committee (Hospices Civils de Lyon, CPP Sud-Est-III). A cohort of STEMI patients who underwent primary percutaneous coronary intervention were included. Plasma samples were collected and stored at –80°C. We assessed sPD-1 levels at 2 time points: discharge from hospital (48 hours after admission) and 1 month. An enzyme-linked immunosorbent assay (R&D Systems) was used for analysis. Patients underwent cardiac magnetic resonance imaging at 1 month in which infarct size and left ventricular ejection fraction (LVEF) were assessed. Clinical events were prospectively collected. Statistical analyses were conducted by using GraphPad Prism version 10.2.0 (GraphPad). The Wilcoxon signed rank test (matched pairs) was used to compare sPD-1 levels at different time points. Comparison of Kaplan-Meier curves for the cumulative incidence of the composite endpoint was performed by using the log-rank test. A *P* value <0.05 was considered statistically significant.

**Figure 1:**
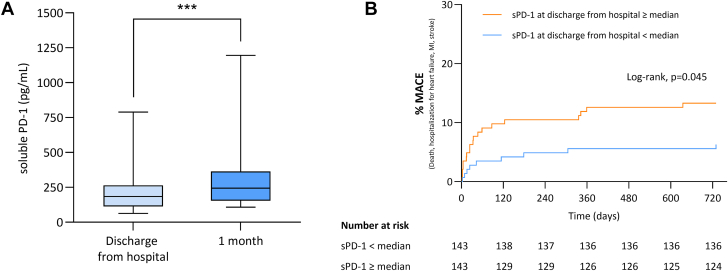
sPD-1 at Discharge From the Hospital and 1 Month After STEMI and MACE (A) Soluble programmed death (sPD)-1 release in a cohort of patients with ST-segment elevation myocardial infarction (STEMI). (B) Cumulative incidence of the composite endpoint (major adverse cardiovascular event [MACE]) according to sPD-1 serum level at discharge from the hospital. Data are represented as median (horizontal bar in box) with 25th to 75th percentiles (vertical box range) and 10th to 90th percentiles (vertical range). ∗∗∗*P* < 0.001.

The mean age of the study cohort was 59 ± 12 years; 53.2% had anterior MI. Median LVEF was 53% (25th to 75th percentiles [Q1-Q3]: 46%-59%), and infarct size was 14.0% of the left ventricle (Q1-Q3: 7.0%-23.2%). Median plasma sPD-1 levels were 183.8 pg/mL (Q1-Q3: 112.5-264.5 pg/mL) at discharge from the hospital with a significant increase 1 month after STEMI (244.4 ng/mL; Q1-Q3: 154.3-364.7 ng/mL; *P* < 0.001) (Figure 1A). No correlation between sPD-1 and infarct size (*r* = 0.02; *P* = 0.75) or LVEF (*r* = –0.04; *P* = 0.55) was found at discharge. Similar results were observed at 1 month. There were 28 major adverse cardiovascular events (MACE) during the 2 years of follow-up (7 MIs, 3 ischemic strokes, 14 hospitalizations for heart failure, and 4 all-cause deaths). Patients with sPD-1 levels greater than or equal to the median value (183.8 pg/mL) were more likely to experience MACE at 2 years after STEMI (HR: 2.2; 95% CI: 1.05-4.6; *P* = 0.045) (Figure 1B). In a multivariate model including age, sex, creatinine kinase peak, and Thrombolysis In Myocardial Infarction flow, sPD-1 level at discharge greater than or equal to the median value was an independent factor of increased risk of adverse events (adjusted HR: 2.3; 95% CI: 1.01-5.1; *P* = 0.048). Similarly, sPD1 levels greater than or equal to the median value at 1 month after STEMI was associated with an increased risk of experiencing MACE in univariable and multivariable models, respectively (HR: 4.0 [95% CI: 1.5-11.1; *P* = 0.02]; adjusted HR: 3.5 [95% CI: 1.1-11.3; *P* = 0.04]).

Myocardial injury after MI leads to the activation of the immune system to promote tissue repair and restore homeostasis. PD-1 is viewed as an immune gatekeeper with studies describing its role in preventing an uncontrolled inflammatory reaction.^3^ We showed, for the first time, that STEMI leads to an increase in sPD1 plasma level between hospital discharge and 1 month. Our preliminary data suggest that sPD1 plasma level in STEMI patients might be an independent prognosis biomarker, reflecting the magnitude of the T-cell inhibition process. These results are in line with previous data exploring the role of immune checkpoint inhibitors in cancer therapies.^4^ It remains uncertain whether the measured sPD-1 was biologically active, including its potential to modulate the PD-1 axis, with attention to associated risks such as induced myocarditis.^5^ Because of the relatively small sample size, low number of events, and the lack of a replication cohort, the findings must be regarded as provisional.
